# Magnetic resonance guided elective neck irradiation targeting individual lymph nodes: A new concept

**DOI:** 10.1016/j.phro.2021.10.006

**Published:** 2021-11-10

**Authors:** Floris C.J. Reinders, Tristan C.F. van Heijst, Joel Mases, Chris H.J. Terhaard, Patricia A.H. Doornaert, Marielle E.P. Philippens, Cornelis P.J. Raaijmakers

**Affiliations:** Department of Radiotherapy, University Medical Centre Utrecht, the Netherlands

**Keywords:** CA, carotid arteries, *D*_mean_, mean dose, ENI, elective neck irradiation, i-ENI, individual non-suspect lymph node elective neck irradiation, LNs, lymph nodes, i-LNs, individual lymph nodes, MRL, magnetic resonance imaging linear accelerator, OAR, organ at risk, OC, oral cavity (OC), PCM, pharynx constrictor muscle, PG, parotid gland, SMG, submandibular gland, Magnetic resonance imaging, Lymph nodes, Radiotherapy, Squamous cell carcinoma of head and neck, Head and neck neoplasms

## Abstract

**Background and purpose:**

Conventional elective neck irradiation (ENI) in head and neck cancer consists of radiotherapy (RT) to the regional lymph node (LN) levels contoured on computed tomography. Hybrid Magnetic Resonance (MR) - RT modalities, such as combined magnetic resonance imaging - linear accelerators (MRLs), might enable new ENI strategies in which individual non-suspect lymph nodes (i-LNs) are targeted. In this treatment planning study, new MR-based strategies targeting i-LNs (i-ENI) were compared to conventional treatment.

**Materials and methods:**

All i-LNs were delineated on MR images of ten retrospectively selected patients with T2-4aN0M0 laryngeal cancer. Three strategies were considered. Strategy A: Conventional ENI delivered with a conventional linear accelerator (35x 1.55 Gy). Strategy B: MRL-based i-ENI (35x 1.55 Gy) to the individual lymph nodes including a background dose to the conventional elective neck volumes (35x 1.03 Gy). Strategy C: Same as Strategy B, but without background dose. In all plans the dose prescription to the primary tumor was 35x 2 Gy. Mean dose (*D*_mean_) reductions in the organs at risk (OAR) were compared using the Wilcoxon signed rank test.

**Results:**

Compared to conventional ENI (strategy A), significant *D*_mean_ reductions of 6.0 Gy and 8.0 Gy were observed in the submandibular glands, of 9.4 Gy and 13 Gy in the carotid arteries and of 9.9 Gy and 19.4 Gy in the thyroid for strategy B and C, respectively. Large inter-patient variations of *D*_mean_ reductions were observed in all OARs.

**Conclusion:**

MRL-based i-ENI is a new promising concept that could reduce the mean dose to OARs in the neck significantly for patients with laryngeal cancer.

## Highlights

• Individual elective lymph nodes can be identified using multiple Dixon T2-weighted turbo spin echo with fat suppression.
• Magnetic Resonance guided individual lymph node irradiation results in lower dose to the organs at risk.
• Especially the submandibular glands, carotid arteries and thyroid can be spared.
• The magnetic field on the magnetic resonance imaging - linear accelerator did not lead to increased skin dose depositions.

## 1 Introduction

For the treatment of regional occult metastases in patients with laryngeal cancer, elective neck irradiation (ENI) to the regional lymph node (LN) levels is prescribed with a radiation dose of 46–55 Gy. The LN levels are based on anatomical borders, as determined on computed tomography (CT) using delineation guidelines [1], and encompass the regions where individual lymph nodes (i-LNs) could be located. Due to the relatively large treatment volumes, ENI is associated with significant morbidity. Long-term complications include xerostomia [2], dysphagia [3], hypothyroidism [4] and carotid stenosis [5]. Over the past decades diagnostic imaging has improved substantially, lowering the detection threshold of small regional tumor deposits. Still, the dose prescription and target selection for ENI has largely remained unchanged [6]. Therefore, in recent years, several studies have been initiated exploring the de-intensification of ENI to reduce the toxicity of radiation therapy (RT) in patients with head and neck cancer (HNC). Some of these studies succeeded in decreasing the total RT dose in ENI to 35–40 Gy, without increasing the regional recurrence (RR) rate [7], [8], [9].

A different approach to reduce RT toxicity for HNC patients could be achieved by reducing the electively treated volumes. In the ideal situation only i-LNs are irradiated instead of large regional LN levels. However, the identification of i-LNs is problematic with conventional CT-based RT planning. In recent years new imaging modalities, including magnetic resonance imaging (MRI), have been introduced and successfully integrated into the RT planning process [10]. With the advent of new MRI techniques it is possible to better visualize soft-tissue structures including (small) i-LNs. This enables a new approach for ENI in which we propose to identify clinically non-suspect i-LNs with MRI and treat them accordingly, which we refer to as individual lymph node treatment in elective neck irradiation (i-ENI). With irradiation of i-LNs, the RT dose to the conventional target volumes can be reduced which, in turn, could result in a lower dose to the organs-at-risk (OARs) and reduced RT toxicity for patients with laryngeal cancer.

i-ENI includes targeting multiple small i-LNs simultaneously. We anticipate that accurate online (i.e. while the patient is on the treatment table) identification and position verification of these small soft tissue structures is difficult and mandates MRI in order to minimize potential set-up errors. Fortunately, performing online MRI position verification is currently available with hybrid MRI-RT modalities, such as combined magnetic resonance imaging - linear accelerators (MRLs).

In this study, two new MR-based i-ENI strategies were compared to conventional ENI in patients with laryngeal cancer. The aim was to explore the potential reduction of RT dose to the OARs.

## 2 Materials and methods

### 2.1 Study designs and patient selection

In this in silico study, all pre-treatment imaging of ten patients with squamous cell carcinoma of the larynx (cT2-4aN0M0) treated at the University Medical Centre (UMC) Utrecht, The Netherlands, between 2016 and 2019, were randomly selected out of an anonymized database. The primary tumor was located at the supraglottic level in four patients while six patients had a tumor located at the glottic level.

### 2.2 CT and MR imaging

During image acquisition for RT planning purposes, patients were immobilized in RT treatment position in the same custom-made 5-points thermoplastic mask. A treatment-planning CT was acquired: slice thickness 3 mm and minimal in-plane resolution was 1x1 mm^2^. MRI scanning was performed on a 3 T MRI scanner, using two flexible receive coils and a posterior receive coil inside the scanner table. The water-only image of the multiple Dixon T2-weighted turbo spin echo (T2 mDixon TSE) scan [11] was used for identification of the i-LNs (slice thickness: 2 mm, in plane resolution: 0.94 × 0.94 mm^2^), such that i-LNs could be separated from the fatty environment they are located in. The MRI scans were co-registered to the treatment-planning CT, based on mutual information, and manually adjusted if necessary.

### 2.3 Definition and delineation of target structures

All target structures were contoured by a radiation oncologist, using the treatment planning CT and MRI scans. The gross tumor volume (GTV) consisted of the primary tumor and was contoured on CT. Subsequently, the clinical target volume of the primary tumor (CTV_p_) was created by adding a 5-mm margin to the GTV in all spatial directions, excluding air and bony tissue [12]. The corresponding primary planning target volume (PTV_p_) was generated by expanding the CTV_p_ with a margin of 3, 4 and 6–8 mm in respectively lateral, ventro-dorsal and cranio-caudal directions [13]. The conventional bilateral elective LN regions (CTV_n_) of LN level II-IV were contoured on the CT according to the guidelines published by the European Organization for Research and Treatment of Cancer (EORTC) [1]. The PTV_n_ was generated by adding a uniform margin of 3 mm to the CTV_n_. All visible i-LNs were identified and delineated (CTV_i-LNs_) on the T2-TSE MRI which were given a margin of 3 mm, according to the conventional margins used for the PTV_n_, to create the PTV_i-LNs_. i-LNs were identified as structures with hyperintense signal inside the conventional nodal neck volumes.

### 2.4 Delineation of OARs

The OARs consisted of the parotid glands (PGs), submandibular glands (SMGs), oral cavity (OC), pharynx constrictor muscles (PCMs), carotid arteries (CA), thyroid and the body contour. All OARs were delineated on CT according to international consensus guidelines [14]. The skin, defined as the most superficial 5 mm of the body contour surface, was contoured as well in order to ascertain possible adverse effects on skin dose due to the static magnetic field inside the MRL. The absolute volume of the skin receiving 35 Gy or higher (*V*_35Gy_) was considered to be clinically relevant [15].

### 2.5 RT strategies

**Fig. 1:**
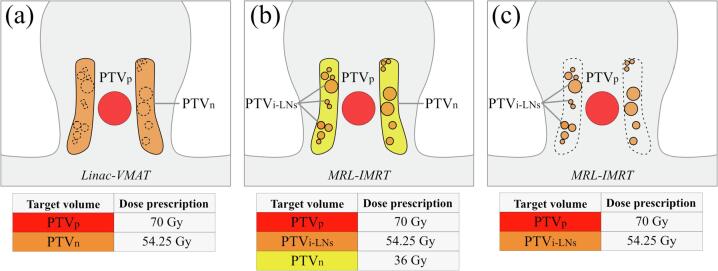
Overview of the three RT strategies (A, B, C), schematically indicated in the coronal plane. The target volumes are displayed per scenario; PTV_p_ (red), PTV_i-LNs_ (orange), and PTV_n_ (yellow). (a) - Strategy A covers conventional ENI on a linear accelerator by VMAT. (b) - Strategy B covers i-ENI with an additional background dose. (c) - Strategy C covers only the individual LNs. (For interpretation of the references to color in this figure legend, the reader is referred to the web version of this article.)

Three different RT strategies were employed (Fig. 1). The primary dose prescribed to the PTV_p_ was 35x 2 Gy = 70 Gy, while varying elective dose prescriptions and RT techniques were applied:

A. Conventional ENI:

35x 1.55 Gy = 54.25 Gy to the conventional bilateral elective LN regions (PTV_n_); RT is performed on a conventional 6 MV linear accelerator (linac) by Volumetric-Modulated Arc Therapy (VMAT).

B. MR-based i-ENI, with background RT dose:

35x 1.55 Gy = 54.25 Gy to the visible non-suspect individual lymph nodes (PTV_i-LNs_), with a ‘background’ RT dose of 35 × 1.05 = 36 Gy to the conventional PTV_n_ excluding the visible nodes; RT is performed on a 7 MV 1.5 T MRL by intensity-modulated RT (IMRT).

C. MR-based i-ENI, no background RT dose:

35x 1.55 Gy = 54.25 Gy was prescribed to the non-suspect individual lymph nodes (PTV_i-LNs_) without a ‘background’ dose prescription; RT is performed on a 7 MV 1.5 T MRL by IMRT.

Strategy A was intended as the clinical standard RT treatment. The new approach of i-ENI on an MRL was explored in strategies B and C. In strategy C, the maximum potential of OAR sparing was aimed at only irradiating i-LNs, while strategy B was introduced to serve as an intermediate approach between strategies A and C. The difference between B and C is the addition of a so-called background dose to the conventional PTV_n_ in strategy B. Theoretically, (very) small i-LNs containing micro-metastasis could be missed on MRI. In order to treat these, a background RT dose of 36 Gy was prescribed in 35 fractions (33 Gy EQD2_(α/β= 10)_).

### 2.6 Treatment planning

The plans were generated on the treatment-planning CT. The primary aim of the treatment planning was to achieve clinically acceptable plans for the three strategies. The volume of the PTVs receiving at least 95% of the prescribed dose (*V*_95%_) was aimed to be 98% or higher. Air inside PTVs was omitted from the structure to ensure sufficient target coverage. Target overdose (*V*_107%_) was set at a maximum of 1% for each PTV (Table 1). Other technical details on the methods used for treatment planning of all strategies can be found in supplementary material 1. Dose distributions and dose volume histograms (DVHs) were generated for each patient and strategy. Plan evaluation was performed by assessing dosimetric parameters in the OARs. The mean dose (*D*_mean_) received by the OARs, and the *V*_35Gy_ in case of the skin, were determined.

**Table 1:** Dosimetric target prescription and OAR constraints used for RT planning of all three strategies. PTV = planning target volume, OAR = organ at risk, V_x%_= relative volume receiving x% of the prescribed RT dose, D_*max*_ = maximum dose, ALARA = As low as reasonably achievable. Soft constraints are recommended but may be higher in individual plans. Hard constraints are mandatory for plan approval.

**All PTVs**	
Target coverage	*V*_95%_ > 98%
Target overdose	*V*_107%_ < 1%
**Soft constraints OARs**	
Parotid glands	*D_mean_* < 20 Gy
Submandibular glands	*D*_mean_ < 39 Gy*
Oral cavity	*D_mean_* < 50 Gy
Pharynx constrictor muscles	*D_mean_* < 35 Gy
Thyroid	ALARA
Unspecified tissue	ALARA
**Hard constraints OARs**	
Carotid arteries	*D_max_* < 70 Gy
Mandible	*D_max_* < 70 Gy
Brain stem	*D_max_* < 55 Gy
Spinal cord	*D_max_* < 50 Gy

*At least one gland.

### 2.7 Statistical analysis

Ordinal variables are reported as absolute values. Continuous variables are reported as median with inter quartile range (IQR). Plan evaluation comparing *D*_mean_ received by the OARs between strategy B vs. strategy A and strategy C vs. strategy A was conducted using Wilcoxon signed-rank test due to the relatively small sample size. All statistical testing was performed with SPSS (Version 25.0). A *p*-value < 0.05 was considered statistically significant.

## 3 Results

**Fig. 2:**
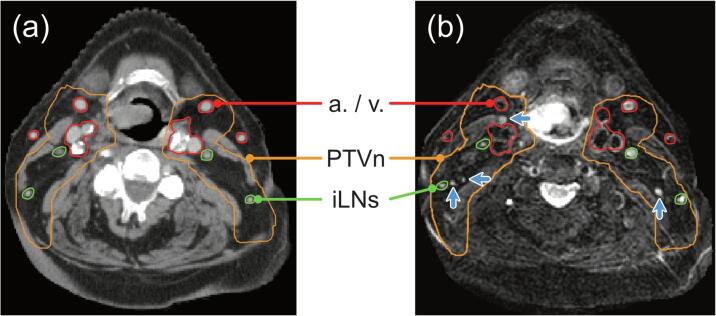
Comparison of lymph node visibility on CT and MRI. Corresponding transverse slices of a planning CT scan (a) and the water-only image of a T2-weighted MRI scan using mDixon fat separation (b). Blood vessels are indicated in red, conventional elective neck volumes (PTV_n_) in orange, and individual elective lymph nodes (i-LNs) in green. The blue arrows in (b) indicate four i-LNs that are not identified on CT, but that were found and contoured on the MRI scan in this slice. (For interpretation of the references to color in this figure legend, the reader is referred to the web version of this article.)

**Fig. 3:**
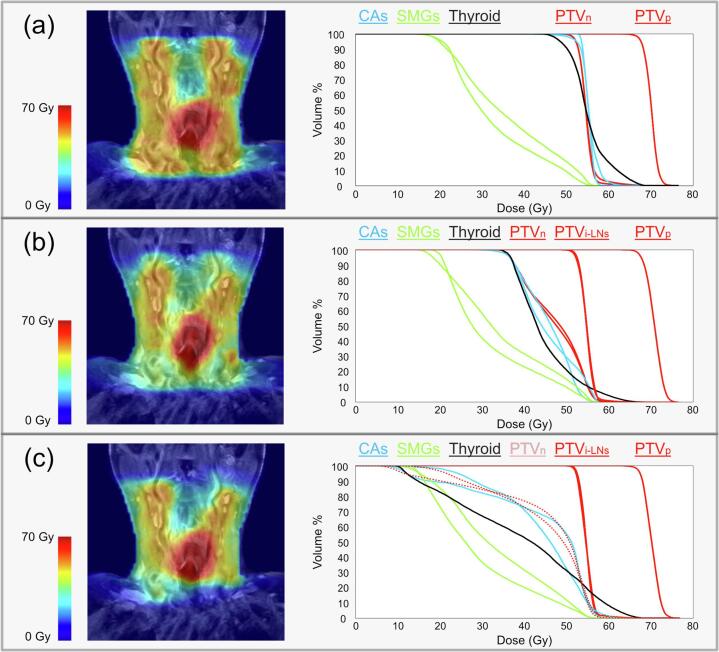
Example of dose maps for strategy A - (a), strategy B - (b), and strategy C - (c), shown as a color wash projected on the same coronal slice of a T2-weighted MRI scan (first column), and corresponding DVHs (second column), for one patient. Abbreviations: CAs = carotid arteries, SMGs = submandibular glands, PTV = planning target volume, PTV_p_ = PTV of primary tumor, PTV_n_ = PTV of conventional LN levels, PTV_i-LNs_ = PTV of i-LNs.

The mean numbers of i-LNs observed on the MR images on the right/left side were 18/17, respectively. Whereas on CT only 12 i-LNs were identified on both the right and left side of the neck (supplementary Table 1). The smallest size of delineated i-LNs on MRI was 3 mm measured over the longitudinal axis in the transversal plane. In Fig. 2, the difference in the conspicuity of i-LNs on CT and MRI is demonstrated. The resulting absolute volumes of PTV_i-LNs_ were 85% smaller compared to the conventional PTV_n_. For all patients clinically acceptable plans were generated for strategies A, B and C in which OAR dose constraints, in terms of maximum dose (*D*_max_) or mean dose (*D*_mean_), were met (Fig. 3, supplementary Tables 2, 3 and 4).

### 3.1 Dose reductions in OARs

MR-based individual lymph node irradiation (i-ENI), with and without background dose (strategies B/C) resulted in significant reductions of *D*_mean_ across all patients in the submandibular glands (-8.5/-10.6 Gy), parotid glands (-2.2/-4.0 Gy), pharynx constrictor muscles (-2.8/-5.6 Gy), carotid arteries (-8.9/-11.8 Gy) and thyroid (-8.7/-18.0 Gy), when compared to conventional treatment (strategy A). Non-significant *D*_mean_ reductions, between strategies B/C and A were found in the oral cavity (+0.4/-3.8 Gy). The absence of the background RT dose in strategy C resulted in an extra *D*_mean_ reduction across all patients in all OARs ranging from −1.8 to −9.3 Gy, compared to strategy B (Table 2). No disadvantageous effects on skin dose were observed due to the magnetic field in the MRL. Actually, compared to conventional elective RT by VMAT (strategy A), MR-based strategies B and C showed an average decrease of −12.2 cm^3^ and –33.0 cm^3^ of skin *V*_35Gy_, respectively (Table 2).

**Table 2:** Dosimetric parameters for all OARs for three RT strategies A, B, and C, as values averaged for all plans. For all OARs but the skin, the *D*_mean_ is displayed; the V_35Gy_ is listed for the skin. For strategies B and C, also the difference compared to strategy A is indicated (B vs. A or C vs. A), as an average difference. Abbreviations: SMGs = submandibular glands, PGs = parotid glands, OC = oral cavity, PCMs = pharynx constrictor muscles, CAs = carotid arteries.

	Strategy a	Strategy b			Strategy c		
OARs	*D*_mean_ Gy (IQR)	*D*_mean_ Gy (IQR)	Difference Gy, b vs. a	*p*-value	*D*_mean_ Gy (IQR)	Difference Gy, c vs. a	*p*-value
SMGs	44.8 (41.4–47.7)	36.3 (32.8–43.4)	−8.5	0.02	34.2 (30.9–42.2)	−10.6	0.01
PGs	15.7 (14.1–16.9)	13.5 (12.3–15.1)	−2.2	0.01	11.7 (10.9–13.6)	−4.0	0.01
OC	13.3 (8.5–17.3)	13.7 (10.7–17.4)	+0.4	0.58	9.5 (8.0–15.0)	−3.8	0.33
PCMs	43.9 (39.7–50.3)	41.1 (36.6–45.8)	−2.8	0.02	38.3 (34.8–48.7)	−5.6	0.01
CAs	55.6 (55.0–56.9)	46.6 (45.2–48.6)	−8.9	0.01	43.8 (39.1–47.2)	−11.8	0.01
Thyroid	53.1 (50.1–56.8)	44.4 (39.3–48.1)	−8.7	0.01	35.2 (25.9–41.8)	−18.0	0.01

	***V*_35Gy_ cm^3^ (IQR)**	***V*_35Gy_ cm^3^ (IQR)**	**Difference cm^3^, b vs. a**	***p*-value**	***V*_35Gy_ cm^3^ (IQR)**	**Difference cm^3^, c vs. a**	***p*-value**

Skin	91.9 (75.3–118.0)	79.7 (63.9–90.2)	−12.2	0.04	58.9 (40.6–62.9)	–33.0	0.01

### 3.2 Inter-patient variation

It was not possible to achieve a reduction of *D*_mean_ in all OARs with strategies B and C, compared to A in every patient. Sparing of the CAs and thyroid was realized in all patients, while the SMGs, PGs, OC and PCMs structures received a slightly higher dose in some of the MR-based plans.

Relatively large variation in *D*_mean_ reductions in the OARs were observed between patients. The differences in *D*_mean_ varied from −12.3 to + 1.6 Gy in the submandibular glands (2 patients received a higher dose in the MR-based plans), from −13.3 to −6.8 Gy in the carotid arteries and from −16.2 to – 6.2 Gy in the thyroid with strategy B vs. A. These variations were even larger for strategy C compared with strategy A. The *D*_mean_ reductions for the other OARs were smaller (Table 2).

## 4 Discussion

Targeting i-LNs facilitated by MRI guidance is a promising new concept. Significant *D*_mean_ reductions were achieved with MR-based i-ENI in the SMGs, PGs, PCMs, CAs and thyroid, compared to conventional treatment. Most notably, average *D*_mean_ reductions>5 Gy were found in the SMGs, CAs and thyroid. In the SMGs however, these reductions were not achieved in all patients. Based on the results of this study we expect the concept of MR-based i-ENI has the potential to reduce RT toxicity for laryngeal patients without compromising the dose in the lymph nodes.

As a result of the *D*_mean_ reductions, advantageous effects on RT-associated toxicity could be expected for patients with laryngeal cancer who are treated with MR-based i-ENI. Based on the organ-specific Normal Tissue Control Probability (NTCP) model for the SMGs [16], the number of patients with salivary flow < 25% of the SMGs 1 year after RT could be expected to decrease by 12% and 16% in case of MR-based i-ENI, with and without additional background RT dose prescription, respectively. For hypothyroidism [17] this reduction amounts to 12% and 22%. Unfortunately no NTCP models are currently available for the CAs; however previous studies revealed that dose reductions in the CAs are associated with less carotid stenosis and cerebrovascular events [5], [18], [19], [20], [21]. These studies imply that the dose reduction could lead to a clinically meaningful reduction in side effects in the majority of our patients.

Previous studies described potentially increased dose depositions at skin-tissue interfaces due to the static magnetic field in the MRL [15], [22]. This could lead to undesired radiation-induced toxicity. In this study no increase of dose in the most superficial 5 mm of the skin (*V*_35Gy_) was observed in the MR-based plans.

On average 18 i-LNs were delineated in the elective neck volumes on each side per patient, varying from 12 to 31 i-LNs. A higher number of i-LNs (approximately 6 additional i-LNs per side) were identified on MR compared to CT. In a pathological study comparable results were described by Pou *et al.* who analyzed 118 elective neck dissections in which on average 21.15 LNs were counted per unilateral neck dissection. Nonetheless, 47.5% of all specimens contained < 18 LNs and 18.6% had even < 10 LNs [23]. The variation of the counted LNs could be due to the natural anatomical variation found in humans.

In the present study, a 3 T MR-scanner was used, as in the radiotherapy simulation phase, for optimal target and LNs identification. Visibility of small i-LNs on the MRL might be problematic since a lower gradient (1.5 T vs 3 T) is applied and no dedicated head and neck coil is available. Therefore, in an ongoing study, we assess the sensitivity of a 1.5 T MRL for individual lymph node identification in comparison to the 3 T MR scanner (the first results are shown in supplementary material 2).

The PTV margins for i-LNs were adopted from the PTV margins of the conventional elective neck volumes and are used to compensate for set-up errors. These PTV margins do not included possible movement of i-LNs during RT treatment. Therefore, a separate study will be performed in which the intrafraction and interfraction movement of i-LNs will be determined with MR. The results from the intrafraction, interfraction and i-LNs visibility studies will also indicate whether practical issues such as on-line delineation procedures and adaptive strategies will limit clinical implementation.

Few i-LNs were found in the cranial and caudal parts of the conventional elective nodal volumes. As a consequence, when no background RT dose was prescribed with i-ENI (strategy C), the elective field sizes were reduced in the cranial and caudal directions. These reductions could partly explain the large *D*_mean_ reductions for the thyroid found in strategy C. The sparing effect in the thyroid was smaller when an additional background RT dose was prescribed to the entire LN volumes (strategy B).

Sparing of the CAs and thyroid was possible for every patient. However, the SMGs could not always be spared and in some plans received a slightly higher *D*_mean_ due to variations between VMAT and IMRT planning. The highest sparing potential was observed in anatomical situations where the distance between the target volumes (primary tumor and/or i-LNs) and the SMGs was the largest.

Other groups investigate de-intensification of ENI as well. Three previously published studies succeeded in decreasing the dose to the elective neck to 36–40 Gy [7], [8] or excluded LN levels [24] without increasing the RR. Other ongoing studies are selecting fewer LN levels based on LN drainage patterns [25] or imaging parameters [26]. Our proposed concept of i-ENI is a different approach in which MRI guidance could enable a more delimited elective target definition, thereby potentially allowing for healthy tissue to be better spared. It is conceivable that two or more de-intensification approaches could be combined in future studies to further reduce RT related toxicity.

The background dose of 33 Gy (EQD2) used in strategy B is based on three considerations. First of all, the background dose is applied in patients who do not have clinical nodal involvement (N0) and therefore have a low probability of having occult metastasis. Secondly, all visible i-LNs and occult metastases in those i-LNs are irradiated with the conventional dose and do not have to be covered by the background dose. Thirdly, the background dose is only needed to cover the treatment of occult metastases not laying inside the visible i-LNs and therefore will be smaller than the smallest i-LN that is detected with MRI. Calculations by van den Bosch et al. [27] showed a regional tumor control probability (TCP) of 94% if patients received ENI with a total dose of 33 Gy (EQD2_(α/β= 10)_), under the assumption that all occult metastases had a diameter smaller than 3 mm. In our study the smallest size of detected i-LNs was 3 mm. In addition to this rationale, we are convinced that a lower background dose is justified than the dose (36–40 Gy EQD2) used in other clinical studies [7], [8] that investigated the de-intensification of ENI, since our background dose is only needed for small occult metastasis (<3 mm) in N0 patients.

Since MR-based i-ENI will have an impact on both patient burden and costs, it is reasonable to select only patients in whom substantial dose reductions in the OARs are expected. For this selection process, a plan comparison for each patient could be performed between different RT strategies. Since plan comparison is a time-consuming process, it could be more efficient to utilize the distance of target areas relative to the OARs as guideline to predict which patients are most likely to benefit from i-ENI.

In patients with laryngeal cancer, significant *D*_mean_ reductions in OARs were observed with MR-based i-ENI compared to conventional treatment. Even with the use of a 36-Gy background RT dose, large *D*_mean_ reductions (>5 Gy) can be achieved in the thyroid and carotid arteries for all patients and in the submandibular glands for a half of these patients. In selected patients, adapting elective treatment to the i-LNs could lead to less salivary gland dysfunction, carotid stenosis (i.e. stroke) and hypothyroidism.

## Declaration of Competing Interest

The authors declare that they have no known competing financial interests or personal relationships that could have appeared to influence the work reported in this paper.
