# Supplementary material for: Magnetic resonance guided elective neck irradiation targeting individual lymph nodes: A new concept
Source: Phys Imaging Radiat Oncol. 2021 Nov 10;20:76–81. doi: 10.1016/j.phro.2021.10.006 (PMC8829887; doi:10.1016/j.phro.2021.10.006)
Supplement: Supplementary material 1 [file mmc1.docx]

**Supplementary material 1**

Planning specifications

Conventional linac planning (strategy A)

Clinical treatment planning software (TPS) Monaco v5.10 (Elekta, Stockholm, Sweden) was employed for strategy A. All VMAT plans consisted of a double arc without table rotation. The Monte Carlo dose calculation algorithm was used to calculate the fluence optimization with 3-mm dose grid and the plan was subsequently segmented. The number of segments and the monitor units per fraction per individual plan can be found in the table below.

MRL planning (strategies B and C)

For strategies B and C, TPS Monaco v5.40 was used which is dedicated for MRL treatment simulations using IMRT. Physical properties of the MRL are accounted for, including the static magnetic field, the cryostat transmission, MRL couch model and MRI receiver coil. All MRL plans comprised of 13 beam angles (200°, 220°, 270°, 290°, 320°, 335°, 0°, 25°, 40°, 70°, 90°, 140°, 160°) with a fixed isocenter. The graphics processing unit (GPU)-based Monte Carlo dose (GPUMCD) calculation algorithm was used for fluence optimization with a 3-mm dose grid and the plan was subsequently segmented. The number of segments and the monitor units per fraction per individual plan can be found in the table below.

|  | **Patient 1** | **Patient 2** | **Patient 3** | **Patient 4** | **Patient 5** | **Patient 6** | **Patient 7** | **Patient 8** | **Patient 9** | **Patient 10** | **MIN** | **MAX** | **Mean** | **SD** |
| --- | --- | --- | --- | --- | --- | --- | --- | --- | --- | --- | --- | --- | --- | --- |
| **VMAT** |  |  |  |  |  |  |  |  |  |  |  |  |  |  |
| Number of segments | 146 | 140 | 159 | 158 | 146 | 118 | 152 | 158 | 162 | 156 | 118 | 162 | 149.50 | 13.11 |
| Monitor Units per fraction | 629.43 | 803.51 | 639.30 | 624.32 | 633.47 | 614.49 | 651.30 | 840.85 | 640.33 | 728.93 | 614.49 | 840.85 | 680.59 | 81.44 |
| **MRL-IMRT i-LNs + background** |  |  |  |  |  |  |  |  |  |  |  |  |  |  |
| Number of segments | 102 | 69 | 57 | 78 | 107 | 102 | 84 | 92 | 89 | 109 | 57 | 109 | 88.90 | 17.13 |
| Monitor Units per fraction | 1052.43 | 721.31 | 604.62 | 831.99 | 1115.62 | 1057.41 | 881.19 | 962.87 | 933.76 | 1101.24 | 604.62 | 1115.62 | 926.24 | 169.10 |
| **MRL-IMRT i-LNs - background** |  |  |  |  |  |  |  |  |  |  |  |  |  |  |
| Number of segments | 68 | 67 | 53 | 66 | 61 | 79 | 44 | 70 | 74 | 75 | 44 | 79 | 65.70 | 10.60 |
| Monitor Units per fraction | 807.52 | 727.35 | 554.41 | 794.99 | 611.60 | 935.41 | 500.20 | 806.13 | 920.70 | 816.28 | 500.20 | 935.41 | 747.46 | 147.82 |
