# Supplementary material for: Magnetic resonance guided elective neck irradiation targeting individual lymph nodes: A new concept
Source: Phys Imaging Radiat Oncol. 2021 Nov 10;20:76–81. doi: 10.1016/j.phro.2021.10.006 (PMC8829887; doi:10.1016/j.phro.2021.10.006)
Supplement: Supplementary Table 1 [file mmc2.docx]

**Supplementary table 1**

| **Patient ID** | **Patient 01** | **Patient 02** | **Patient 03** | **Patient 04** | **Patient 05** | **Patient 06** | **Patient 07** | **Patient 08** | **Patient 09** | **Patient 10** | **MIN** | **MAX** | **Mean** | **SD** |
| --- | --- | --- | --- | --- | --- | --- | --- | --- | --- | --- | --- | --- | --- | --- |
| **INDIVIDUAL LYMPH NODES** |  |  |  |  |  |  |  |  |  |  |  |  |  |  |
| Number of nodes Right (MRI) | 14 | 14 | 20 | 31 | 12 | 17 | 18 | 17 | 19 | 27 | 12 | 31 | 18.9 | 5.93 |
| Number of nodes Right (CT) | 11 | 9 | 15 | 18 | 10 | 11 | 14 | 8 | 7 | 18 | 7 | 18 | 12.1 | 3.96 |
| Number of nodes Left (MRI) | 16 | 16 | 16 | 17 | 15 | 13 | 15 | 21 | 17 | 28 | 13 | 28 | 17.4 | 4.25 |
| Number of nodes Left (CT) | 12 | 9 | 11 | 14 | 14 | 10 | 12 | 16 | 7 | 17 | 7 | 17 | 12.2 | 3.12 |
| TOTAL (MRI) | 30 | 30 | 36 | 48 | 27 | 30 | 33 | 38 | 36 | 55 | 27 | 55 | 36.3 | 8.86 |
| TOTAL (CT) | 23 | 18 | 26 | 32 | 24 | 21 | 26 | 24 | 14 | 35 | 14 | 35 | 24.3 | 6.13 |

Supplementary table 1: Number of elective lymph nodes counted in all patients
