# Supplementary material for: Magnetic resonance guided elective neck irradiation targeting individual lymph nodes: A new concept
Source: Phys Imaging Radiat Oncol. 2021 Nov 10;20:76–81. doi: 10.1016/j.phro.2021.10.006 (PMC8829887; doi:10.1016/j.phro.2021.10.006)
Supplement: Supplementary Table 2 [file mmc3.docx]

**Supplementary table 2**

| **Strategy A: Conventional ENI (VMAT)** | | | | | | | | | | | | | | |
| --- | --- | --- | --- | --- | --- | --- | --- | --- | --- | --- | --- | --- | --- | --- |
| **Patient ID** | **Patient 01** | **Patient 02** | **Patient 03** | **Patient 04** | **Patient 05** | **Patient 06** | **Patient 07** | **Patient 08** | **Patient 09** | **Patient 10** | **MIN** | **MAX** | **Mean** | **SD** |
| **TARGET COVERAGE** | (%) | (%) | (%) | (%) | (%) | (%) | (%) | (%) | (%) | (%) |  |  |  |  |
| PTV_p_ V95% (70.00 Gy) | 98.21 | 98.25 | 98.31 | 98.33 | 99.00 | 98.26 | 99.30 | 98.75 | 98.86 | 98.84 | 98.21 | 99.30 | 98.61 | 0.39 |
| PTV_n_ R V95% (54.25 Gy) | 98.81 | 98.84 | 99.74 | 99.25 | 99.02 | 99.15 | 98.79 | 97.88 | 98.81 | 98.58 | 97.88 | 99.74 | 98.89 | 0.48 |
| PTV_n_ L V95% (54.25 Gy) | 99.17 | 99.35 | 99.65 | 98.00 | 98.85 | 99.21 | 98.58 | 98.10 | 97.18 | 99.18 | 97.18 | 99.65 | 98.73 | 0.76 |
| ***D_mean_* OAR** | (Gy) | (Gy) | (Gy) | (Gy) | (Gy) | (Gy) | (Gy) | (Gy) | (Gy) | (Gy) |  |  |  |  |
| ***D_mean_* Salivary OAR** |  |  |  |  |  |  |  |  |  |  |  |  |  |  |
| SG R (Gy) | 47.25 | 42.39 | 42.58 | 46.07 | 39.05 | 57.19 | 41.98 | 32.65 | 48.90 | 45.40 | 32.65 | 57.19 | 44.35 | 6.46 |
| SG L (Gy) | 47.84 | 44.88 | 41.86 | 46.97 | 38.55 | 47.13 | 43.61 | 35.58 | 47.59 | 46.53 | 35.58 | 47.84 | 44.05 | 4.20 |
| SG R+L (Gy) | 47.55 | 43.64 | 42.22 | 46.52 | 38.80 | 52.16 | 42.80 | 34.12 | 48.25 | 45.97 | 34.12 | 52.16 | 44.20 | 5.13 |
| PG R (Gy) | 14.39 | 19.18 | 17.42 | 14.62 | 15.75 | 23.03 | 19.13 | 14.28 | 18.05 | 15.43 | 14.28 | 23.03 | 17.13 | 2.80 |
| PG L (Gy) | 11.34 | 14.23 | 14.18 | 13.62 | 15.52 | 18.08 | 15.96 | 15.54 | 13.96 | 12.72 | 11.34 | 18.08 | 14.52 | 1.87 |
| PG R+L (Gy) | 12.87 | 16.71 | 15.80 | 14.12 | 15.64 | 20.56 | 17.55 | 14.91 | 16.01 | 14.08 | 12.87 | 20.56 | 15.82 | 2.15 |
| ***D_mean_* Vascular OAR** |  |  |  |  |  |  |  |  |  |  |  |  |  |  |
| CA R (Gy) | 56.68 | 58.74 | 56.30 | 55.37 | 56.52 | 63.74 | 54.66 | 55.08 | 56.96 | 54.55 | 54.55 | 63.74 | 56.86 | 2.73 |
| CA L (Gy) | 55.02 | 57.87 | 56.43 | 54.66 | 54.51 | 58.75 | 54.36 | 55.31 | 54.31 | 55.20 | 54.31 | 58.75 | 55.64 | 1.55 |
| CA R+L (Gy) | 55.85 | 58.31 | 56.37 | 55.02 | 55.52 | 61.25 | 54.51 | 55.20 | 55.64 | 54.88 | 54.51 | 61.25 | 56.25 | 2.05 |
| ***D_mean_* Swallow OAR** |  |  |  |  |  |  |  |  |  |  |  |  |  |  |
| PCMs | 40.22 | 51.49 | 39.75 | 49.89 | 47.47 | 66.00 | 48.39 | 39.43 | 40.18 | 34.27 | 34.27 | 66.00 | 45.71 | 9.06 |
| OC | 11.00 | 24.04 | 6.31 | 8.77 | 21.52 | 15.94 | 15.41 | 12.98 | 13.63 | 7.66 | 6.31 | 24.04 | 13.73 | 5.77 |
| **D_mean_ other OAR** |  |  |  |  |  |  |  |  |  |  |  |  |  |  |
| Thyroid | 47.62 | 50.70 | 56.09 | 60.51 | 48.18 | 58.83 | 52.94 | 55.32 | 53.34 | 51.77 | 47.62 | 60.51 | 53.53 | 4.24 |
| **V35Gy skin** | (cc) | (cc) | (cc) | (cc) | (cc) | (cc) | (cc) | (cc) | (cc) | (cc) |  |  |  |  |
| Skin 5mm (body - 5mm) | 65.90 | 140.30 | 91.60 | 114.80 | 89.20 | 100.70 | 127.40 | 92.10 | 72.10 | 76.30 | 65.90 | 140.30 | 97.04 | 24.17 |

Supplementary table 2: Target coverage and mean dose (D_mean_) in the OARs in strategy A (conventional elective lymph node irradiation).
PTV = planning target volume, PTVp= planning target volume of gross tumor volume, PTV_n_ =planning target volume of elective LN levels, OAR = organ at risk, SG = submandibular gland, PG = parotid gland, CA = carotid artery, PCMs = pharynx constrictor muscles, OC = oral cavity, R = right, L =left, Min = minimum value, Max =maximum value, SD = standard deviation, V35Gy = volume that receives 35 Gy or more, cc= cubic centimeter.
