# Supplementary material for: Magnetic resonance guided elective neck irradiation targeting individual lymph nodes: A new concept
Source: Phys Imaging Radiat Oncol. 2021 Nov 10;20:76–81. doi: 10.1016/j.phro.2021.10.006 (PMC8829887; doi:10.1016/j.phro.2021.10.006)
Supplement: Supplementary Table 3 [file mmc4.docx]

**Supplementary table 3**

| **Strategy B: i-ENI with a background dose (IMRT-MRL)** | | | | | | | | | | | | | | |  |
| --- | --- | --- | --- | --- | --- | --- | --- | --- | --- | --- | --- | --- | --- | --- | --- |
| **Patient ID** | **Patient 01** | **Patient 02** | **Patient 03** | **Patient 04** | **Patient 05** | **Patient 06** | **Patient 07** | **Patient 08** | **Patient 09** | **Patient 10** | **Min** | **MAX** | **Mean** | **SD** | |
| **TARGET COVERAGE** | (%) | (%) | (%) | (%) | (%) | (%) | (%) | (%) | (%) | (%) |  |  |  |  | |
| PTV_p_ V95% (70.00 Gy) | 98.64 | 99.11 | 98.49 | 98.01 | 98.59 | 99.25 | 98.62 | 98.75 | 98.33 | 98.71 | 98.01 | 99.25 | 98.65 | 0.35 | |
| PTV_i-LNs_ R (54.25 Gy) | 99.14 | 99.79 | 99.54 | 99.13 | 99.06 | 99.56 | 99.53 | 98.91 | 99.36 | 97.99 | 97.99 | 99.79 | 99.20 | 0.51 | |
| PTV_i-LNs_ L (54.25 Gy) | 99.19 | 99.88 | 99.78 | 98.86 | 97.90 | 98.76 | 98.39 | 98.03 | 98.38 | 98.28 | 97.90 | 99.88 | 98.75 | 0.69 | |
| PTV_n_ R V95% (36.00 Gy) | 99.87 | 99.94 | 99.87 | 99.83 | 99.75 | 99.44 | 99.01 | 99.79 | 99.48 | 99.83 | 99.01 | 99.94 | 99.68 | 0.29 | |
| PTV_n_ L V95% (36.00 Gy) | 99.79 | 99.95 | 99.95 | 99.08 | 99.53 | 99.59 | 98.76 | 99.78 | 99.54 | 99.90 | 98.76 | 99.95 | 99.59 | 0.39 | |
| ***D_mean_* OAR** | (Gy) | (Gy) | (Gy) | (Gy) | (Gy) | (Gy) | (Gy) | (Gy) | (Gy) | (Gy) |  |  |  |  | |
| ***D_mean_* Salivary OAR** |  |  |  |  |  |  |  |  |  |  |  |  |  |  | |
| SG R (Gy) | 44.54 | 44.79 | 40.37 | 36.21 | 37.14 | 56.57 | 34.31 | 31.62 | 36.92 | 34.00 | 31.62 | 56.57 | 39.65 | 7.36 | |
| SG L (Gy) | 41.11 | 45.58 | 28.04 | 37.79 | 27.14 | 49.80 | 30.06 | 34.35 | 35.00 | 39.25 | 27.14 | 49.80 | 36.81 | 7.43 | |
| SG R+L (Gy) | 42.83 | 45.19 | 34.21 | 37.00 | 32.14 | 53.19 | 32.19 | 32.99 | 35.96 | 36.63 | 32.14 | 53.19 | 38.23 | 6.83 | |
| PG R (Gy) | 13.58 | 18.47 | 17.46 | 11.24 | 12.60 | 14.59 | 14.41 | 11.77 | 15.18 | 11.52 | 11.24 | 18.47 | 14.08 | 2.46 | |
| PG L (Gy) | 11.74 | 16.90 | 13.53 | 9.79 | 12.09 | 15.28 | 13.20 | 15.71 | 11.28 | 13.04 | 9.79 | 16.90 | 13.26 | 2.19 | |
| PG R+L (Gy) | 12.66 | 17.69 | 15.50 | 10.52 | 12.35 | 14.94 | 13.81 | 13.74 | 13.23 | 12.28 | 10.52 | 17.69 | 13.67 | 2.00 | |
| ***D_mean_* Vascular OAR** |  |  |  |  |  |  |  |  |  |  |  |  |  |  | |
| CA R (Gy) | 45.84 | 52.36 | 47.89 | 43.25 | 46.60 | 59.04 | 42.66 | 45.44 | 49.77 | 44.24 | 42.66 | 59.04 | 47.71 | 4.96 | |
| CA L (Gy) | 45.47 | 50.56 | 47.32 | 44.99 | 46.25 | 46.83 | 39.84 | 45.58 | 43.91 | 49.53 | 39.84 | 50.56 | 46.03 | 2.97 | |
| CA R+L (Gy) | 45.66 | 51.46 | 47.61 | 44.12 | 46.43 | 52.94 | 41.25 | 45.51 | 46.84 | 46.89 | 41.25 | 52.94 | 46.87 | 3.35 | |
| ***D_mean_* Swallow OAR** |  |  |  |  |  |  |  |  |  |  |  |  |  |  | |
| PCMs | 42.47 | 51.60 | 36.92 | 43.88 | 39.69 | 62.08 | 43.41 | 35.78 | 38.96 | 32.43 | 32.43 | 62.08 | 42.72 | 8.61 | |
| OC | 11.16 | 19.19 | 9.41 | 13.75 | 14.93 | 21.56 | 13.72 | 10.25 | 16.84 | 10.84 | 9.41 | 21.56 | 14.17 | 4.03 | |
| **D_mean_ other OAR** |  |  |  |  |  |  |  |  |  |  |  |  |  |  | |
| Thyroid | 33.50 | 41.48 | 46.88 | 51.60 | 40.18 | 51.75 | 36.77 | 44.74 | 44.12 | 45.61 | 33.50 | 51.75 | 43.66 | 5.88 | |
| **V35Gy skin** | (cc) | (cc) | (cc) | (cc) | (cc) | (cc) | (cc) | (cc) | (cc) | (cc) |  |  |  |  | |
| Skin 5mm (body - 5mm) | 53.60 | 154.60 | 81.00 | 91.50 | 66.60 | 89.80 | 86.10 | 78.40 | 64.20 | 63.10 | 53.60 | 154.60 | 82.89 | 28.21 | |

Supplementary table 3: Target coverage and mean dose (D_mean_) in the OARs in strategy B (MRL-based elective neck irradiation to the elective lymph nodes with a background dose). PTV = planning target volume, PTVp= planning target volume of gross tumor volume, PTV_i-LNs_= planning target volume of individual elective lymph nodes, PTV_n_ =planning target volume of elective LN levels, OAR = organ at risk, SG = submandibular gland, PG = parotid gland, CA = carotid artery, PCMs = pharynx constrictor muscles, OC = oral cavity, R = right, L =left, Min = minimum value, Max =maximum value, SD = standard deviation, V35Gy = volume that receives 35 Gy or more, cc= cubic centimeter.
