# Supplementary material for: Magnetic resonance guided elective neck irradiation targeting individual lymph nodes: A new concept
Source: Phys Imaging Radiat Oncol. 2021 Nov 10;20:76–81. doi: 10.1016/j.phro.2021.10.006 (PMC8829887; doi:10.1016/j.phro.2021.10.006)
Supplement: Supplementary Table 4 [file mmc5.docx]

| **Strategy C: i-ENI without a background dose (IMRT-MRL)** | | | | | | | | | | | | | | |
| --- | --- | --- | --- | --- | --- | --- | --- | --- | --- | --- | --- | --- | --- | --- |
| **Patient ID** | **Patient 01** | **Patient 02** | **Patient 03** | **Patient 04** | **Patient 05** | **Patient 06** | **Patient 07** | **Patient 08** | **Patient 09** | **Patient 10** | **Min** | **MAX** | **Mean** | **SD** |
| **TARGET COVERAGE** | (%) | (%) | (%) | (%) | (%) | (%) | (%) | (%) | (%) | (%) |  |  |  |  |
| PTV_p_ V95% (70.00 Gy) | 98.13 | 98.02 | 98.26 | 98.74 | 99.08 | 98.26 | 98.1 | 98.48 | 98.12 | 99.43 | 98.02 | 99.43 | 98.46 | 0.48 |
| PTV_i-LNs_ R (54.25 Gy) | 98.95 | 99.1 | 99.15 | 99.03 | 99.42 | 99.49 | 97.67 | 99.37 | 99.3 | 99.39 | 97.67 | 99.49 | 99.09 | 0.53 |
| PTV_i-LNs_ L (54.25 Gy) | 98.7 | 98.97 | 99.62 | 98.34 | 99.38 | 99.06 | 97.11 | 98.71 | 99.21 | 98.1 | 97.11 | 99.62 | 98.72 | 0.73 |
| ***D_mean_* OAR** | (Gy) | (Gy) | (Gy) | (Gy) | (Gy) | (Gy) | (Gy) | (Gy) | (Gy) | (Gy) |  |  |  |  |
| ***D_mean_* Salivary OAR** |  |  |  |  |  |  |  |  |  |  |  |  |  |  |
| SG R (Gy) | 43.46 | 43.87 | 40.49 | 27.29 | 34.01 | 56.67 | 29.10 | 28.84 | 35.17 | 33.57 | 27.29 | 56.67 | 37.25 | 9.04 |
| SG L (Gy) | 40.40 | 41.89 | 29.53 | 36.81 | 27.65 | 48.43 | 25.83 | 33.05 | 31.65 | 36.50 | 25.83 | 48.43 | 35.17 | 7.03 |
| SG R+L (Gy) | 41.93 | 42.88 | 35.01 | 32.05 | 30.83 | 52.55 | 27.47 | 30.95 | 33.41 | 35.04 | 27.47 | 52.55 | 36.21 | 7.49 |
| PG R (Gy) | 15.47 | 15.71 | 16.59 | 8.87 | 11.46 | 13.64 | 14.15 | 10.93 | 12.53 | 11.53 | 8.87 | 16.59 | 13.09 | 2.45 |
| PG L (Gy) | 10.50 | 14.65 | 12.69 | 8.87 | 10.56 | 9.22 | 12.24 | 11.89 | 11.41 | 9.57 | 8.87 | 14.65 | 11.16 | 1.78 |
| PG R+L (Gy) | 12.99 | 15.18 | 14.64 | 8.87 | 11.01 | 11.43 | 13.20 | 11.41 | 11.97 | 10.55 | 8.87 | 15.18 | 12.12 | 1.91 |
| ***D_mean_* Vascular OAR** |  |  |  |  |  |  |  |  |  |  |  |  |  |  |
| CA R (Gy) | 38.31 | 46.62 | 40.60 | 43.68 | 46.41 | 54.93 | 34.18 | 43.79 | 48.32 | 45.58 | 34.18 | 54.93 | 44.24 | 5.71 |
| CA L (Gy) | 36.86 | 46.75 | 38.65 | 41.36 | 44.27 | 42.61 | 37.66 | 44.12 | 39.06 | 52.13 | 36.86 | 52.13 | 42.35 | 4.71 |
| CA R+L (Gy) | 37.59 | 46.69 | 39.63 | 42.52 | 45.34 | 48.77 | 35.92 | 43.96 | 43.69 | 48.86 | 35.92 | 48.86 | 43.29 | 4.45 |
| ***D_mean_* Swallow OAR** |  |  |  |  |  |  |  |  |  |  |  |  |  |  |
| PCMs | 41.04 | 49.82 | 36.87 | 37.57 | 39.01 | 64.32 | 48.33 | 32.06 | 35.73 | 26.65 | 26.65 | 64.32 | 41.14 | 10.65 |
| OC | 9.75 | 18.22 | 9.31 | 11.21 | 8.07 | 19.57 | 6.33 | 7.97 | 13.95 | 8.67 | 6.33 | 19.57 | 11.31 | 4.51 |
| **D_mean_ other OAR** |  |  |  |  |  |  |  |  |  |  |  |  |  |  |
| Thyroid | 12.13 | 25.5 | 36.84 | 51.89 | 33.46 | 39.9 | 29.43 | 38.92 | 26.03 | 47.32 | 12.13 | 51.89 | 34.14 | 11.55 |
| **V35Gy skin** | (cc) | (cc) | (cc) | (cc) | (cc) | (cc) | (cc) | (cc) | (cc) | (cc) |  |  |  |  |
| Skin 5mm (body - 5mm) | 45.30 | 110.70 | 62.70 | 58.40 | 43.10 | 63.63 | 62.00 | 59.30 | 27.70 | 33.10 | 27.70 | 110.70 | 56.59 | 22.97 |

**Supplementary table 4**

Supplementary table 4: Target coverage and mean dose (D_mean_) in the OARs in strategy C (MRL-based elective neck irradiation to the elective lymph nodes without a background dose). PTV = planning target volume, PTVp= planning target volume of gross tumor volume, PTV_i-LNs_= planning target volume of individual elective lymph nodes, PTV_n_ =planning target volume of elective LN levels, OAR = organ at risk, SG = submandibular gland, PG = parotid gland, CA = carotid artery, PCMs = pharynx constrictor muscles, OC = oral cavity, R = right, L =left, Min = minimum value, Max =maximum value, SD = standard deviation, V35Gy = volume that receives 35 Gy or more, cc= cubic centimeter.
