# Supplementary material for: Magnetic resonance guided elective neck irradiation targeting individual lymph nodes: A new concept
Source: Phys Imaging Radiat Oncol. 2021 Nov 10;20:76–81. doi: 10.1016/j.phro.2021.10.006 (PMC8829887; doi:10.1016/j.phro.2021.10.006)
Supplement: Supplementary material 2 [file mmc6.docx]

**Supplementary material 2**

*
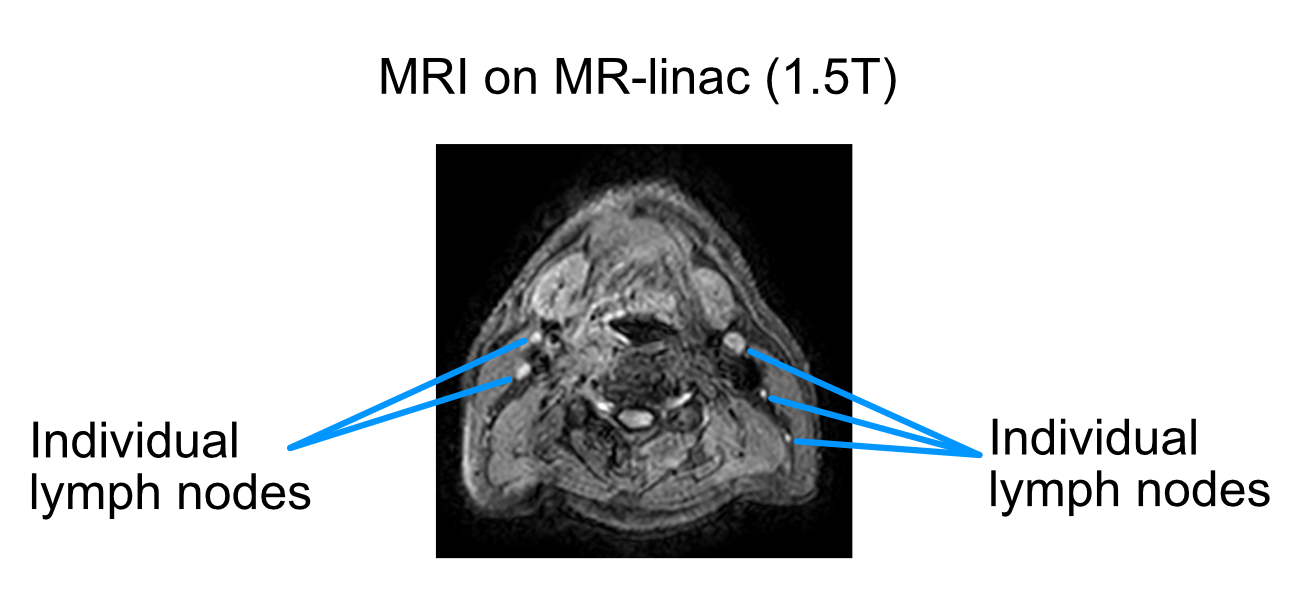
*

Figure: Imaging of a head and neck cancer patient on the 1.5T MR-linac. Individual lymph nodes are clearly visible on both sides of the neck.
